# SkinNet-16: A deep learning approach to identify benign and malignant skin lesions

**DOI:** 10.3389/fonc.2022.931141

**Published:** 2022-08-08

**Authors:** Pronab Ghosh, Sami Azam, Ryana Quadir, Asif Karim, F. M. Javed Mehedi Shamrat, Shohag Kumar Bhowmik, Mirjam Jonkman, Khan Md. Hasib, Kawsar Ahmed

**Affiliations:** ^1^ Department of Computer Science (CS), Lakehead University, Thunder Bay, ON, Canada; ^2^ College of Engineering, IT and Environment, Charles Darwin University, Darwin, NT, Australia; ^3^ Department of Software Engineering, Daffodil International University, Dhaka, Bangladesh; ^4^ Department of Computer Science and Engineering, Ahsanullah University of Science & Technology, Dhaka, Bangladesh; ^5^ Department of Electrical and Computer Engineering, University of Saskatchewan, Saskatoon, SK, Canada

**Keywords:** skin cancer, principle component analysis, image processing, ROI, Otsu thresholding, machine learning, deep learning

## Abstract

Skin cancer these days have become quite a common occurrence especially in certain geographic areas such as Oceania. Early detection of such cancer with high accuracy is of utmost importance, and studies have shown that deep learning- based intelligent approaches to address this concern have been fruitful. In this research, we present a novel deep learning- based classifier that has shown promise in classifying this type of cancer on a relevant preprocessed dataset having important features pre-identified through an effective feature extraction method.

Skin cancer in modern times has become one of the most ubiquitous types of cancer. Accurate identification of cancerous skin lesions is of vital importance in treating this malady. In this research, we employed a deep learning approach to identify benign and malignant skin lesions. The initial dataset was obtained from Kaggle before several preprocessing steps for hair and background removal, image enhancement, selection of the region of interest (ROI), region-based segmentation, morphological gradient, and feature extraction were performed, resulting in histopathological images data with 20 input features based on geometrical and textural features. A principle component analysis (PCA)-based feature extraction technique was put into action to reduce the dimensionality to 10 input features. Subsequently, we applied our deep learning classifier, SkinNet-16, to detect the cancerous lesion accurately at a very early stage. The highest accuracy was obtained with the Adamax optimizer with a learning rate of 0.006 from the neural network-based model developed in this study. The model also delivered an impressive accuracy of approximately 99.19%.

## 1 Introduction

Skin cancer is a type of cancer that originates in the tissues of the skin and is due to the development of abnormal growth of cells that can invade or spread to other parts of the body. It was the fourth most common cancer in the year 2020 (1). Skin cancer embodies a particular challenge for estimating incidence for several reasons. There are multiple subtypes of skin cancer, and non-melanoma skin cancer is often not tracked by cancer registries (2). Registrations of skin cancer are often incomplete because most cases are successfully treated *via* surgery or ablation. Some countries do not have cancer registries, regions of some countries have few or no records, records in countries suffering war or other disruption are bound to be incomplete, and some people with cancer do not consult a physician. Due to these factors, it is likely that the reported global incidence of skin cancer is an underestimate. Australia and New Zealand have the highest rates of reported skin cancer (Australia 33.6 per 100,000 and New Zealand 33.3 per 100,000) (3), followed by the Scandinavian countries in Europe. The apparent reason behind this high rate is that the majority of skin cancers are caused by exposure to UV radiation in sunlight. Skin cancers are most frequently found on the head and neck of human (4). In the Southeast Asian region, this type of cancer is less common according to the Global Cancer Observatory (5, 6). As skin cancer develops on the outside of the body and is a visible type of disease, it can be examined by a dermatologist very early. Detecting skin cancer lesions at an early stage significantly reduces morbidity, decreases healthcare costs, and improves patient survival rate (7). Dermoscopy is a non-invasive examination technique for the visual investigation of the surface structure of the skin. This detection using dermoscopy is undeniably higher than individual observation-based detection, but the accuracy of the diagnostic depends on the training of the dermatologist. The inadequate number of trained dermatologists (8) makes it hard to accurately diagnose cancerous lesions at an early stage.

### 1.1 The major contributions of the paper

The research goal of this paper is to create an artificial intelligent system that identifies skin lesions accurately at an early stage. It is expected that this ultimately will prevent metastases and (9) and reduce mortality. The key issues are maintained in the following manner:

1. All 3,297 images were selected from the source dataset (10). Selected images were cleaned and made noise-free followed by a number of preprocessing methods before the final dataset is compiled for this research.

2. To produce the impactful feature set, the PCA feature selection technique was employed to reduce the dimensionality into half (10 features).

3. To detect the skin lesions, our produced SkinNet-16 classifier, a generalized model with no overfitting, is based on a convolution neural network (CNN) and deployed to achieve the desired goal.

## 2 Literature review

Ameri (11) proposed a skin cancer detection system in 2020 using a deep convolutional neural network (CNN). He used the HAM10000 dermoscopy image database, which contains 3,400 images including melanoma and non-melanoma lesions. Deep CNN was developed to classify the images into two classes: benign and malignant. No lesion segmentation or feature extraction techniques were used. Instead, the raw images were directly used as the input of the CNN. A classification accuracy of 84% was achieved using these raw images. Yu et al. (12) proposed an automated method for recognizing melanoma skin cancer, deploying deep convolutional neural networks (CNNs) using the ISBI 2016 Skin Lesion Analysis toward Melanoma Detection Challenge. They claimed that their proposed method was more accurate than existing architectures because their network contained more than 50 layers. Their network was able to acquire richer and more discriminative features which eventually resulted in a more effective performance. In this two-stage framework, they applied the fully convolutional residual network (FCRN) for skin lesion segmentation and very deep residual networks for classification. The uniqueness of their work was that they worked with limited training data, but a substantially deeper network than other authors, resulting in a classification accuracy of 85.5%. Their work provided some evidence that a two-stage framework with segmentation can achieve better results than direct manipulation of the dermoscopic images. Andre Esteva et al. (13) performed a classification of skin lesions using deep convolutional neural networks. They trained the CNN using a dataset of 129,450 clinical images using only pixels and disease labels as inputs. They then tested its performance against 21 board-certified dermatologists on biopsy-proven clinical images. It was demonstrated that Artificial Intelligence systems might be capable of classifying skin cancer at a level of competence comparable to dermatologists. The accuracy was 96% for carcinoma images, 94% for melanoma images, and 91% for melanoma dermoscopic images 91%. The sensitivity *vs*. specificity curve for the CNN was promising, but the rate of false positive and false negative was still too high to ignore. Jinnai et al. (14) developed a skin cancer classification system for pigmented skin lesions using deep learning. A total of 5,846 clinical images from 3,551 patients were used, and faster region-based CNN (FRCNN) was used as the classifier. For six-class classification, the accuracy of FRCNN was 86.2%, and for two-class classification (benign or malignant), the accuracy was 91.5%. In both cases, FRCNN performed better than other methods and dermatologists. Boman and Volminger (15) proposed a deep convolutional neural network model for the classification of skin cancer in 2018. They evaluated the performance of the CNN based on melanoma versus solar lentigo and melanoma versus seborrheic keratosis. They primarily used the data from the ISIC Dermoscopic Archive dataset of 23,647 images and downloaded an additional 16,826 images from DermQuest, 4,336 images from the Dermatology Atlas, and 1,948 images from the websites DermaAmin, Dermoscopy Atlas, Global Skin Atlas, Hellenic Dermatological Atlas, Medscape, Regional Derm, Skinsight, and the pH2 database. The accuracy was evaluated with both a 16-way classification and a three-way classification. The best accuracy achieved was 91% for the binary classification of seborrheic keratosis versus basal comparison result. This indicated that for binary comparison an acceptable accuracy can be obtained by applying deep learning classifiers. In 2020, Rehan Ashraf et al. (16) proposed a transfer learning-assisted framework based on an intelligent region of interest (ROI) approach for skin cancer detection. Previous deep learning-based methods had used complete images for feature learning which can result in a lack of performance in terms of discriminative feature extraction. An ROI-based approach helps to identify discriminative features as the images contain only that region to train the system. To extract ROIs from the images, they used an improved k-mean algorithm. They subsequently applied a convolutional neural network (CNN)-based transfer learning model with data augmentation for ROI images. Seventy-seven percent of the data were used for training and the remaining 23% as testing data. The ROI-based approach resulted in an accuracy of 97.9% for their first dataset and 97.4% for the second dataset, demonstrating that the ROI approach outperformed previous methods using complete images (global features) for classification. Region of interest (ROI) detection in dermoscopic images was proposed by Goyal et al. (17) in 2018 in order to achieve data augmentation. They deployed CNN (Faster-RCNN) and proposed the use of two object localization meta-architectures for ROI skin lesion detection in dermoscopic images. The performance of their skin localization methods proved to be superior to other segmentation methods for skin lesions. ROI detection not only has the potential for enhancing the quality of the dataset but also can improve the accuracy of lesion localization. The ROI localization in dermoscopic images and the application of FRCNN (Faster-RCNN) Inception V2 to the ISBI-2017 testing dataset resulted in an accuracy of 94.5% and recall of 94.3% outperforming other models. FRCNN also performed best on other previously unseen datasets in terms of precision and recall, establishing its validity. In 2020, Ali et al. (18) proposed a novel fuzzy method-based multilayer perceptron (F-MLP) system for the detection of irregularity in the skin lesion’s border to aid the early identification of melanomas as border irregularity is one of the important signs of skin cancer. Artificial neural networks (ANNs) or multilayer perceptrons (MLPs) have been shown to perform well in supervised learning tasks, although they can be affected by the way the weights are updated during the learning process which can sometimes degrade the performance of the network when applied to test data. To reduce the effect of ambiguous inputs on the learning process, they proposed a fuzzy multilayer perceptron (F-MLP) that takes the ambiguity of the inputs into consideration. Their proposed approach outperformed most current classification methods, in particular its standard neural network counterpart. Using F-MLP, they obtained the best result with an 80:20 training–testing data ratio, resulting in an accuracy of 95.2%. However, the drawback of this proposed fuzzy neural network was that training the network was much more time-consuming. Fujisawa et al. (19) described an automatic deep learning-based skin cancer classification in mid-2019. They used the dataset ILSVR2012 containing 1.2 million images within 1,000 classes. They also showed that useful feature extraction can drastically improve the model’s performance and efficiency. The classification algorithm that yielded the best result for them using the selected feature values was a deep learning-based convolutional neural network (CNN), as it can learn and automatically determine what features are important for classification from the training image set. For 14-class classification, the CNN model resulted in a 75% accuracy, and for two-class classification in an accuracy of 92%, which surpassed that of board-certified dermatologists. An automatic skin cancer detection system from dermoscopic images were proposed by Seyed Mohammad Alizadeh et al. (20) by combining convolutional neural networks and texture features. The datasets they used were ISIC 2016, ISIC 2019, and PH^2^. After preprocessing of the images using the DullRazor algorithm (21), texture features were extracted and their dimension was reduced using kernel principal component analysis (kPCA) for improving the classification performance in the feature extraction-based phase, and their proposed network and the VGG-19-two CNN models were employed to classify images in the CNN phase. These two methods were fed into their ensemble approach, and the final result was obtained by comparing the results of these two methods. This automated system achieved 85.2%, 96.7%, and 97.5% accuracy respectively for three datasets. Maad M. Mijwil (22) analyzed more than 24,000 skin cancer images with the help of the CNN (ConvNet) model applying three architectures, InceptionV3, ResNet, and VGG19, for classifying benign and malignant types. The author employed datasets containing high-resolution images obtained from the ISIC archive between 2019 and 2020. The best-performed InceptionV3 architecture achieved a diagnostic accuracy of 86.90% outperforming the other architectures. Another DCNN model, named lesion classification network (LCNet), was proposed by Ranpreet Kaur et al. (23) to classify malignant and benign melanoma, where they used dermoscopic images from International Skin Imaging Collaboration datastores (ISIC 2016, ISIC2017, and ISIC 2020). For three different datasets, they managed to obtain 81.41%, 88.23%, and 90.42% accuracy, respectively. However, they did not rely on any extensive preprocessing operations and extraction of lesion features using ROI, which was responsible for this relatively reduced accuracy. Using ISIC datasets, Hatice Catal Reis et al. (24) also developed a deep learning-based convolutional neural network (CNN) model to detect benign and malignant lesions, but they incorporated International Skin Imaging Collaboration HAM10000 images (ISIC 2018), ISIC 2019, and ISIC 2020 datasets. This model was developed based on the Inception module used in GoogleNet architecture, and it used fewer parameters and fewer medical images to make the diagnostic time shorter. Nevertheless, more detailed diagnostic results could have been obtained by improving the segmentation study with meta-heuristic algorithms and graph methods. This lightweight model achieved accuracies of 94.59%, 91.89%, and 90.54% respectively for three datasets. Bechelli et al. (25) performed a binary classification to identify the benign and malignant classes of skin cancer from dermoscopic images using machine learning and deep learning images. For the study, two datasets were used, i.e., ISIC archive and HAM10000. For the classification task using machine learning algorithms, LR, LDA, KNN, CART, and GNB algorithms were performed. A mean prediction result was calculated based on maximum diversity, average prediction, and best performance. The deep learning model used in the study was customized by embedding Xception, VGG16, and ResNet50. Later, the models were modified to achieve improved accuracy. The study uses several performance matrix such as accuracy, precision, recall, F-score, FPR, and ROC curve to evaluate prediction results. The ensemble machine learning method shows a precision score of 0.79 and an f-score of 0.70. Subsequently, after modification, VGG16, ResNet50 and Xception shows f-scores of 0.69, 0.61, and 0.50, respectively.

## 3 Dataset

This study employs two publicly available datasets. First is the dataset which is retrieved from Kaggle repositories (26). The dataset origins from the ISIC archive. It is composed of two different types of skin cancer images: benign skin moles and malignant skin moles. A total of 3,297 benign and malignant histopathological images are examined for this research, where the number of benign images is around 1,800, while malignant images are approximately 1,497. All the introduced RGB format images are in 224 × 224 pixels. The second dataset used is the HAM10000 obtained from the Kaggle archive that originates in the Harvard Dataverse (27). The dataset contains seven types of skin lesion classes and a total of 10015 dermatoscopic images. The seven lesions can be grouped into benign and malignant classes for binary classification. In the lesion belonging in the benign group are dermatofibroma (df) with 115 images, benign keratosis-like lesions (bkl) with 1,099 images, vascular lesions (vasc) with 142 images, and melanocytic nevi (nv) with 6,705 images. Similarly, the lesion belonging in the malignant group are basal cell carcinoma (bcc), melanoma (mel), and actinic keratoses and intraepithelial carcinoma/Bowen’s disease (akiec) with 514, 1,113, and 327 images, respectively. The HAM10000 dataset is a highly imbalanced dataset. The near-miss algorithm (28) is employed to balance the dataset. The algorithm randomly eliminates data from the class with high data to balance the dataset. The final balanced dataset has a total of 1,954 data in each benign and malignant class.

## 4 Methodology

### 4.1 Image preprocessing

**Figure 1:**
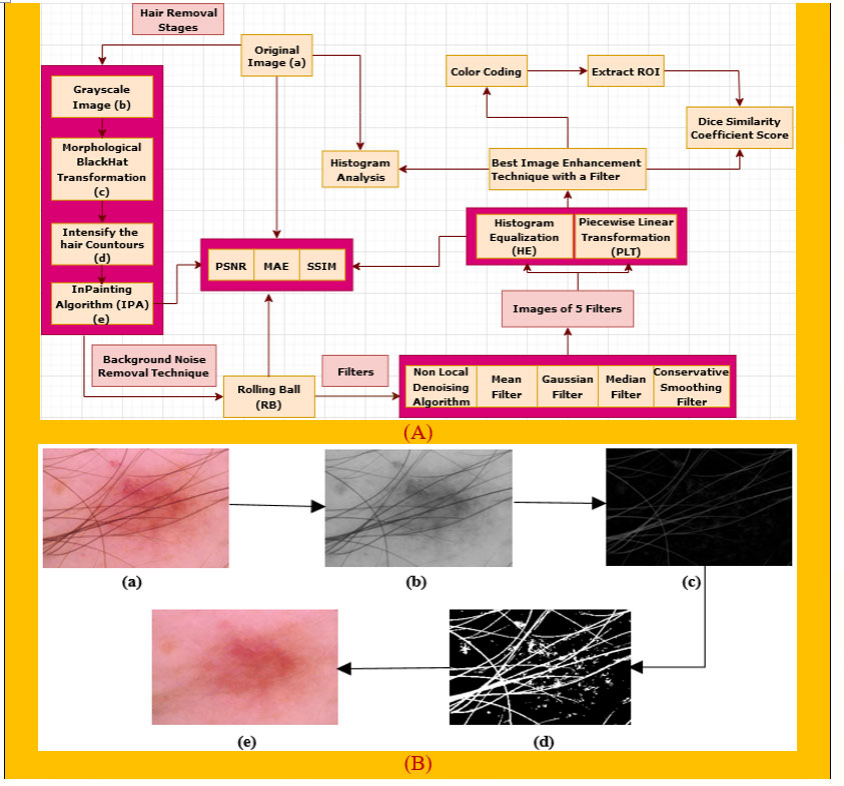
**(A)** Preprocessing technique of our proposed system. **(B)** The DHR process diagram, consisting of the **(a)** original image, **(b)** grayscale image, **(c)** morphological BlackHat operation, **(d)** intensification of the hair contours, and **(e)** Inpainting algorithm.

Preprocessing is a crucial step of image processing (29) in order to obtain accurate outcomes. As there are hairs in a number of the images which could interfere with accurate classification, a digital hair removal (DHR) algorithm (30) is applied next. Next, we apply the rolling ball technique (31) in order to remove background noise. Subsequently, these five well-known filters, namely, non-local means denoising (NLMD) algorithm (32), mean filter (MF) (33), median filter (MDF) (34), Gaussian Filter (GF) (35), and conservative smoothing filter (CSF) (36), are used on the rolling ball image one after another to reduce the unnecessary spots. Thirdly, the image enhancement techniques of histogram equalization (HE) (37) and piecewise linear transformation (PLT) (38) are applied to all the filters. We then picked our best image enhancement technique with a filter based on various types of assessments, namely, PSNR, MAE, SSIM, and histogram analysis, to ensure that the image quality has not been reduced. Finally, color coding (39), a technique for clearer image visualization, is done on our selected image and then the region of interest (ROI) (40) is extracted to show the cancerous lesion of this particular image. Apart from these, the Dice coefficient similarity score (DCS) of our selected image as well as the ROI image (41) is also calculated to evaluate the correctness of our preprocessing techniques, which is famous for comparing the original image with the processed image to forecast the accuracy. **Figure 1A** gives an overview of the preprocessing process.

#### 4.1.1 Hair removal

As artifacts can be the root cause of poor results, removal of the main artifact for skin cancer detection, hairs, is essential. We remove hairs (42) from our images by applying the DHR algorithm (30). This consists of four steps: Grayscale, Morphological BlackHat transformation, creating the mask for InPainting, and the InPainting algorithm.

##### 4.1.1.1 Grayscale image

Grayscale images, as their name suggests, are images containing only shades of gray and no other colors. Grayscale is defined as a range of monochromatic shades (43) from black to white. The luminance value of each pixel, which can also be described as the brightness or intensity, is used to convert the images to gray scale as measured on a scale from black (intensity = 0) to white (intensity = full). For RGB digital images, each pixel has three separate luminance values for red, green, and blue. With the help of cv2.cvtColor () (44), a method of Python OpenCV, we converted our original images to grayscale images.

##### 4.1.1.2 Morphological BlackHat operation

Morphological image processing encompasses a range of image processing techniques that deal with the shape (or morphology) of features in an image. We apply the BlackHat morphological operation to find hair contours with respect to direction. Morphological operations are applied to shrink or enhance some image regions through opening, closing, erosion, and dilation. The BlackHat transformation finds the difference between the closing of the input image and the input image itself (45). BlackHat operators are more suited for grayscale images. Mathematically, it can be represented as in equations (1) and (2).


(1) (A•B)−A [in terms of dilatation and erosion]

(2) [(A⊕B)Θ B]−A,[further break it down]

where A is the input image matrix and B the kernel matrix (46). This transformation is used to enhance image components for which the structuring element is larger as well as components which are darker than their surroundings (47). For hair detection, a structuring element of size 23 × 23 is utilized for this morphological operation.

##### 4.1.1.3 Intensify the hair contours

The output from BlackHat Morphological operation results in image with variations of grayscale intensity. To increase the contrast of the hair regions (47), a binary thresholding is applied; see equation (3).


(3) $$\text{I}\cdot \left(\text{x},\text{y}\right)=\{\begin{array}{l} 1,\\ 0, \end{array}\begin{array}{r} if\;I\left(x,y\right)>threshold\\ otherwise \end{array}$$

h resulting image enhances the hair contours.

##### 4.1.1.4 Inpainting algorithm

The Inpainting algorithm refers to the reconstruction of small missing and damaged portions of images. This activity consists of filling in the missing areas or modifying the damaged ones in a way that is not detectable to an observer who is not familiar with the original images. For missing or damaged areas, one can only hope to produce a plausible rather than an exact reconstruction. Therefore, in order for an inpainting algorithm to be reasonably successful, the regions to be inpainted must be locally small. A user-provided mask specifies the portions (48) of the input image to be retouched, and the algorithm treats the input image as three separate channels (R, G, and B). Let Ω be a small area to be inpainted, and let ∂Ω be its boundary. The simplest version of the algorithm consists of initializing Ω by clearing its color information and repeatedly convolving the region to be inpainted with a diffusion kernel. As the diffusion process is iterated, the inpainting progresses from ∂Ω into Ω. This algorithm uses a weighted average kernel that only considers contributions from the neighbor pixels (i.e., it has a zero weight at the center of the kernel). Algorithm 1 (49) describes this process:

**Algorithm 1:** Inpainting Algorithm.

BEGIN initialize Ω; FOR (iter =0; iter < num_iteration; iter++) convolve masked regions with kernel; ENDFOR END

Intersections between Ω and high-contrast edges are the only places where anisotropic diffusion is required, and such regions usually account for a small percentage of the total area. The outputs of each stage of the DHR process for one image are shown in **Figure 1B** for a better understanding of the explanation above.

##### 4.1.2 Rolling ball algorithm

The rolling ball algorithm (31) is a well-known tool to correct non-uniform brightness, especially in medical images. This algorithm estimates the background intensity of a grayscale image in the case of uneven exposure. It is frequently used in biomedical image processing and was first proposed by Stanley R. Sternberg in 1983 (50). This algorithm has successfully been applied to medical images plotted as a 3D surface, with the pixel value of the image being the surface height. A ball of a user-defined radius is rolled over the backside of the surface creating a background surface and subtracting this background surface from the original image removes large spatial variations of the background intensities. The rolling ball can be explained (7) according to the following way.

**Algorithm 2:** Rolling the Ball.

BEGIN FUNCTION roll_ball (ball, array): WHILE y in range(-radius, height + radius) DO next_line_to_write_in_cache <- (y + radius) % ball_width next_line_to_read <- y + radius IF next_line_to_read < height THEN src <- next_line_to_read * width dest <- next_line_to_write_in_cache * width cache [dest:dest + width] <- pixels [src:src + width] p <- next_line_to-read * width FOR x in range(width) DO pixels[p] <– float (‘inf’) p += 1 ENDFOR ENDIF ENDWHILE END

**Figure 2:**
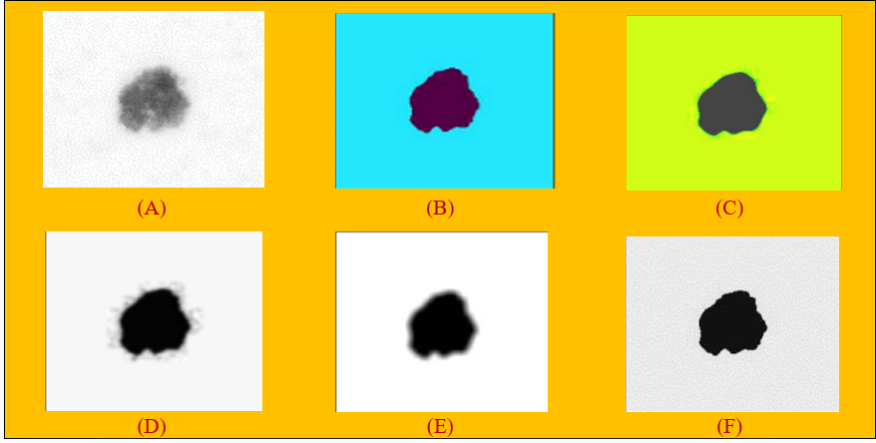
**(A)** Applying rolling ball noise removal technique. **(B)** Non-local means denoising algorithm. **(C)** Median filter on images. **(D)** Gaussian filter on images. **(E)** Mean filter on images. **(F)** Conservative smoothing filter on images.

Algorithm 2 provides the details of how the ball is rolled over the surface of the image. The variables next_line_to_read and next_line_to_write_in_cache are used by the ball to read each pixel in the image, and if the intensity value of the pixel (i.e., height) is greater, then the pixel location is read and stored in the variable “src”. After identifying the location, it is stored in “cache” and the process is repeated till all the pixel values are identified and stored in the cache. To obtain the image “array”, a python program applying the “NumPy” library is used. After applying the rolling ball technique to the hair removal images, the output in **Figure 2A** was obtained.

#### 4.1.3 Image quality improved after hair removal and rolling ball approaches

**Table 1** compares the original image with the hair removed and inpainted image and the rolling ball image based on three significant variables. Here, the rolling ball performs best in two out of three techniques while the values of Original and Hair removed image were quite similar.

**Table 1:** The values of PSNR, MAE, and SSIM of an image.

Images	PSNR (dB)	MAE (dB)	SSIM (dB)
Original	36.2	3.12	0.92
HR image	36.74	2.88	0.93
Rolling ball	42.27	0.83	0.97

1. PSNR = Peak signal-to-noise ratio (between 30 and 50 dB) (7)

2. MAE = Mean Absolute Error (values closer to zero are the better) (7)

3. SSIM = Structural Similarity Index (Range from −1 to +1 and equals 1 for identical images) (7)

#### 4.1.4 Explanations of image filters

Several filters were applied to the image obtained with the rolling ball noise removal technique. They are explained as follows.

##### 4.1.4.1 Non-local means denoising algorithm

The non-local means (NLM) algorithm has been widely used in the field of image processing because of its excellence. Antoni Buades proposed the NLM algorithm originally in 2005 (32, 51). A so-called image block is a square neighborhood centered on a certain pixel point. Let the contaminated image have a gray value of v (i) at pixel i and a filtered gray estimate of NL (v) (i). For any pixel i, the filtered NL (v) (i) can be obtained by computing a weighted average of the pixels in similar neighborhoods of the image; see equations (4-5) (32):


(4) NL(v)(i)=∑(i,j)v(j)

where i is the entire image space; the weighting factor ω (i, j) is the degree of influence of pixel j on pixel i, as shown below:


(5) $$\omega \left(\text{I},\text{j}\right)=\frac{1}{\text{C}\left(\text{I}\right)}{\text{e}}^{\frac{{∥\text{v}\left({\text{N}}_{\text{i}}\right)-\left({\text{N}}_{\text{j}}\right)∥}_{2,\text{a}}^{2}}{{\text{h}}^{2}}}$$

The NLM filtering algorithm shown in **Figure 2B** performs image denoising by calculating the similarity of pixel points in image blocks.

##### 4.1.4.2 Median filter

The median filter is one of the most popular and efficient spatial filters, and it is simple to implement. Although the fundamental drawback of median filtering is blurring the image, it can preserve the edges while simultaneously suppressing the noise (33). Specifically, this filter depicted in **Figure 2**) supplants a pixel by the median value of all pixels in a sliding window. Mathematically, it can be defined as equation (6).


(6) $$\hat{f}\left(x,y\right)=\begin{array}{c} median\left\{g\left(s,t\right)\right\}\\ \begin{array}{cc} \left(x,t\right) & \in S_{xy} \end{array} \end{array}$$

##### 4.1.4.3 Gaussian filter

Gaussian filter is a filter known for its blurring and noise suppressing (35). This filter is a 2D convolution operator with the weights selected pursuant to the shape of Gaussian function (31). The function is defined in equation 7 and a graphical representation is shown in **Figure 2D**:


(7) $$\text{g}\left(\text{x},\text{y}\right)=\frac{1}{\text{M}}\sum \text{f}\left(\text{x},\text{y}\right)\text{ exp}\left[-\left({\left(\text{x}-1\right)}^{2}+{\left(\text{y}-\text{j}\right)}^{2}\right)2\sigma^{2}\right]\left(\text{i},\text{j}\right)\epsilon \text{S}$$

where g(x, y) is the Gaussian distribution, σ is the standard deviation of the distribution, and S is every pixel set in the neighborhood.

M, defined in equation 8.


(8) $$\text{M}=\sum \text{exp}\left[-\left({\left(\text{x}-\text{i}\right)}^{2}+{\left(\text{y}-\text{j}\right)}^{2}\right)2\sigma^{2}\right]$$

This equation defines the set of pixels and corresponding weights of S.

##### 4.1.4.4 Mean filter

Mean filters have a simpler structure compared to median filters. They replace the value of every pixel in an image with the mean (“average”) value of its neighbors (33). This has the effect of eliminating pixel values which are unrepresentative of their surroundings. It is illustrated in **Figure 2E**.

Mean filtering is usually thought of as a convolution. Like other convolutions, it is based around a kernel, which represents the shape and size of the neighborhood to be sampled when calculating the mean. The mean filter is usually used to suppress the small details in an image and also bridge the small gaps that exist in the lines or curves. The mean filter is defined in equation 9.


(9) $$\text{g}\left(\text{i},\text{j}\right)=\frac{1}{\text{M x N}}\sum \text{f}\left(\text{m},\text{n}\right)$$

where m = 1, 2… M and n = 1, 2… N and S is the neighborhood defined by the filter mask of the point f (i, j), centered at point f (i, j).

##### 4.1.4.5 Conservative smoothing filter

Conservative smoothing is a noise reduction technique (36) that gets its name from the fact that it uses a simple, fast-filtering algorithm that sacrifices noise suppression power in order to preserve high spatial frequency details (e.g., sharp edges) in an image. It is explicitly designed to eliminate isolated pixels of exceptionally low or high pixel intensity. It can be used to remove short-range variability in an image, effectively acting to smooth the image. This algorithm operates by calculating the minimum and maximum neighboring values surrounding a grid cell. If the cell at the center of the kernel is greater than the calculated maximum value, it is replaced with the maximum value in the output image. Similarly, if the cell value at the kernel center is less than the neighboring minimum value, the corresponding grid cell in the output image is replaced with the minimum value. The result is shown in **Figure 2F**.

#### 4.1.5 Image enhancement techniques

All the processed images by filters were taken for the purpose of image enhancement. In this context, two supportive approaches, namely, histogram equalization (HE) and piecewise linear transformation (PLT), have been applied to all the images of the dataset to make a set of 3,297 images for each filter after applying an image enhancement technique. As we considered five image filters, we were supposed to get 5 * 3,297 images for a particular enhancement technique; however, we selected the PSNR, MAE, and SSIM values of a filter. The image selection process is completed after implementing both of these enhancement techniques. The explanations to these two techniques are described as follows.

##### 4.1.5.1 Histogram equalization

Histogram equalization (HE) is a technique (37) for adjusting image intensities to enhance the contrast. Let f be a given image represented as an mr by mc matrix of integer pixel intensities ranging from 0 to L − 1. L is the number of possible intensity values, often 256. Let p denote the normalized histogram of with a bin for each possible intensity (see equation 10).


(10) $$p_{n}=\frac{\text{number of pixel with intensity n}}{\text{total number of pixels}}$$

where, n = 0, 1, …, L – 1 and the histogram equalized image g is defined in equation 11.


(11) $$g_{i,j}=\text{floor}\left(\text{L}-1\right)\sum_{n=0}p_{n})$$

where floor () rounds down to the nearest integer. Algorithm 3 takes x as an input signal and generates h as an output of histogram of images (52).

**Algorithm 3:** Histogram Equalization.

**BEGIN** 1. [r,c] = size(x); 2. FOR I = 1:r 3. FOR j = 1:c 4. Hist {x[i,j]} = Hist {x[i,j]} + 1 5. ENDFOR 6. ENDFOR 7. For g = 1:Gmax 8. Hist [g] = Hist [g]/(M*N) 9. ENDFOR **END**

**Figure 3:**
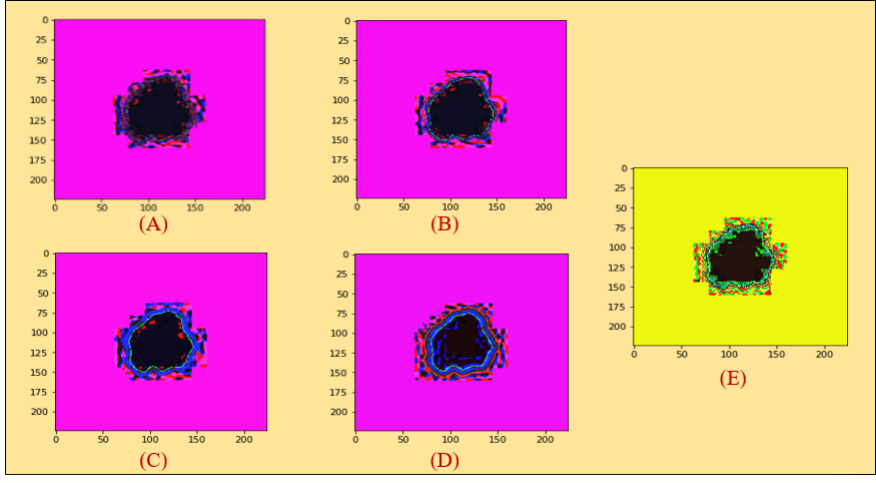
**(A)** Histogram equalization on the non-local means denoising algorithm. **(B)** Histogram equalization on the median filter. **(C)** Histogram equalization on the Gaussian filter. **(D)** Histogram equalization on the mean filter. **(E)** Histogram equalization on the conservative filter.

**Figure 3A** illustrates the non-local means denoising algorithm based on the histogram equalization.

The result of the median filter based on histogram equalization is shown in **Figure 3B**.

In the same way, we demonstrated experimented results in **Figure 3C** on Gaussian filter based on the Histogram Equalization.

In the same manner, experimented results in **Figure 3D** on mean filter are shown based on histogram equalization.

For the conservative filter, we showed the experimented results in **Figure 3E** on it based on histogram equalization.

##### 4.1.5.2 Comparison between filters after using histogram equalization image enhancement 1.1.1.1 technique

**Table 2** shows the output of the previously mentioned filters after applying histogram equalization. Here, the mean filter (MF) provides a better result on five images in terms of all the criteria, while the other filter values are comparatively low.

**Table 2:** Assessments on PSNR, MAE, and SSIM values for five different images of HE and PLT.

HE							PLT				
Images	Image quality assurance	NLMD	MF	GF	MDF	CSF	NLMD	MF	GF	MDF	CSF
Image 4	PSNR (dB)	39.8	41.95	40.3	40.36	40.3	41.65	42.38	40.43	41.86	41.65
	MAE (dB)	4.8	1.53	3.31	4.3	3.8	1.6	0.71	1.42	1.32	1.59
	SSIM (dB)	0.90	0.95	0.92	0.90	0.90	0.95	0.97	0.94	0.96	0.95
Image 7	PSNR (dB)	37.28	43.13	37.14	38.42	31.47	42.8	44.2	43.21	42.90	33.04
	MAE (dB)	4.39	0.66	4.45	3.24	11.09	0.70	0.42	0.51	0.61	5.71
	SSIM (dB)	0.91	0.98	0.92	0.92	0.89	0.97	0.99	0.98	0.98	0.96
Image 17	PSNR (dB)	38.55	39.76	38.55	39.04	36.33	40.18	42.78	41.51	40.72	39.84
	MAE (dB)	1.71	1.18	1.72	1.74	3.93	1.77	0.77	1.15	1.33	1.75
	SSIM (dB)	0.92	0.96	0.92	0.93	0.91	0.96	0.99	0.98	0.97	0.96
Image 38	PSNR (dB)	39.63	42.97	41.44	40.79	38.17	40.77	44.58	41.44	42.5	41.93
	MAE (dB)	1.66	0.62	0.81	1.11	2.79	1.18	0.43	1.2	1.03	1.53
	SSIM (dB)	0.94	0.99	0.98	0.96	0.94	0.95	0.99	0.98	0.99	0.96
Image 56	PSNR (dB)	38.61	41.63	39.89	40.13	39.24	41.62	43.54	41.99	42.24	38.14
	MAE (dB)	4.99	3.18	4.31	3.82	3.93	0.96	0.51	0.68	0.73	2.73
	SSIM (dB)	0.88	0.94	0.90	0.91	0.89	0.97	0.99	0.98	0.98	0.94

##### 4.1.5.3 Piecewise linear transformation

PLT (38) is a spatial domain method that is used for image enhancement. It is applied to increase the dynamic range of gray levels in the image (see Algorithm 4).

**Algorithm 4:** Piecewise Linear Transformation.

**BEGIN** 1. (a) start from the boundary 2. (i) let τ1∈ M be completely labeled 3. (ii) find the unique σ1∈M such that τ1∈ ϵ1 4. (b) start from a ray 5. (i) let σ1∈M have precisely one completely labeled facet τ1 6. (ii) pivoting step: find σ1∈M, σ1=! σ0 such that τ1∈ σ1 7. FOR i = 1,2,3,… 8. (a) IF τ1 is the only completely labeled facet of ϵ1 THEN stop (ray termination) 9. (b) ELSE piecewise linear step: find the other completely labeled facet τi+1 of σi 10. ENDIF 11. (c) if τi+1 ∈ M THEN stop (boundary termination) 12. (d) ELSE pivoting step: find σi+1∈ M, σi+1=! ϵi such that τi+1 ∈ σi+1 13. ENDIF 14. ENDFOR END

**Figure 4:**
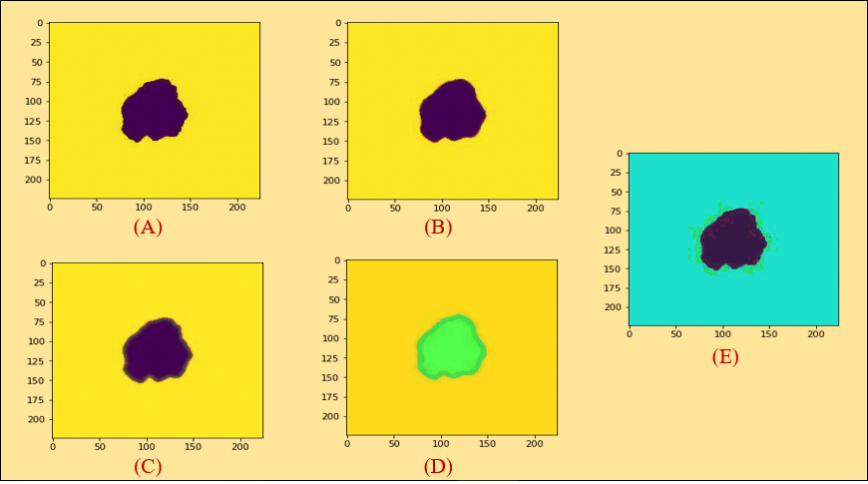
**(A)** Piecewise linear transformation on the non-local means denoising algorithm. **(B)** Piecewise linear transformation on the median filter. **(C)** Piecewise linear transformation on the Gaussian filter. **(D)** Piecewise linear transformation on the mean filter. **(E)** Piecewise linear transformation on the conservative filter.

The result of the non-local means denoising based on PLT is shown in **Figure 4A**.

**Figure 4B** displays the result of the median filter based on piecewise linear transformation.

**Figure 4C** shows the result of the Gaussian filter based on piecewise linear transformation.

The result of the on mean filter based on piecewise linear transformation is shown in **Figure 4D**.

Finally, the results of conservative filter based on piecewise linear transformation are demonstrated in **Figure 4E**.

##### 4.1.5.4 Comparison of different filters after using piecewise linear transformation

**Table 2** compares five types of filters after piecewise linear transformation. Here again, the values of mean filter (MF) are the highest in all the criteria. The overall promising results have been demonstrated based on the randomly chosen images to select the most appropriate filters.

After executing various significant assessments, namely, PSNR, MAE, and SSIM, on these two selected enhancement approaches with the introduced filters, the best values were received from piecewise linear transformation with the mean filter.

#### 4.1.6 Region-of-interest detection with color coding

Color coding (39) is regarded as a process of image visualization that allows the user to gain a deep insight into the structure of the image thoroughly. The use of color for encoding information can greatly improve the observer’s understanding of the information depicted by the image. It is desirable to detect potential targets or regions of interest (ROIs) within various kinds of medical images (53) to ensuring accuracy of the final prediction. These ROIs can also be used to control intelligent region-of-interest-based image compression algorithms, and to direct further analysis for target identification and recognition (40).

**Figure 5:**
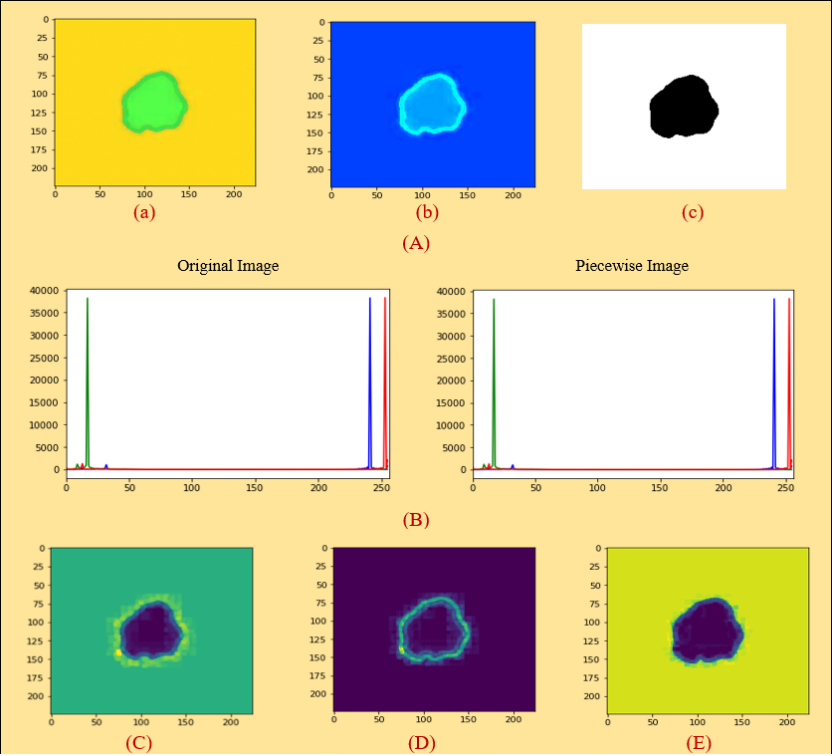
**(A) (a)** Original image (PLT on mean filter), **(b)** color coding, **(c)** ROI image, **(B)** histogram analysis between an original and a PLT image, **(C)** erosion, **(D)** dilation, **(E)** morphological gradient.

At first, we took the selected images as an original image in **Figure 5A a**. We then did color coding on the images of **Figure 5A a** and received an image as shown in **Figure 5A b**. Subsequently, we extracted the cancerous lesion of this image in **Figure 5A c** with the help of Otsu’s thresholding (54, 55) which is used to find a good threshold value by maximizing the variance between objects and background for getting better insight.

#### 4.1.7 A comparison between original and processed image based on histogram analysis

A histogram shows the distribution of the data to assess (7) the central tendency, variability, and shape. A histogram for a quantitative variable divides the range of the values into discrete classes and then counts the number of observations falling into each class interval. Histogram analysis is applied here to evaluate the similarities between an original and a processed piecewise image. Looking at **Figure 5B**, it can be seen that the histogram analysis of the original images is very similar to the histogram analysis of the piecewise images.

#### 4.1.8 Dice coefficient score

The Dice similarity score (41, 56) is computed to compare the original image with the processed image (see equation 12).


(12) $$\text{DSC = }\frac{2*\text{Area of Overlap}}{\text{Total Number of Pixels in both Images}}$$

The area of overlap between an original image and a processed image is estimated, and the Dice coefficient is calculated between two binary images. The Dice similarity coefficient is always between 0 and 1 where 1 means that the two images are identical. We applied this technique to region of interest (ROI) and PLT.

### 4.2 Feature extraction

**Figure 6:**
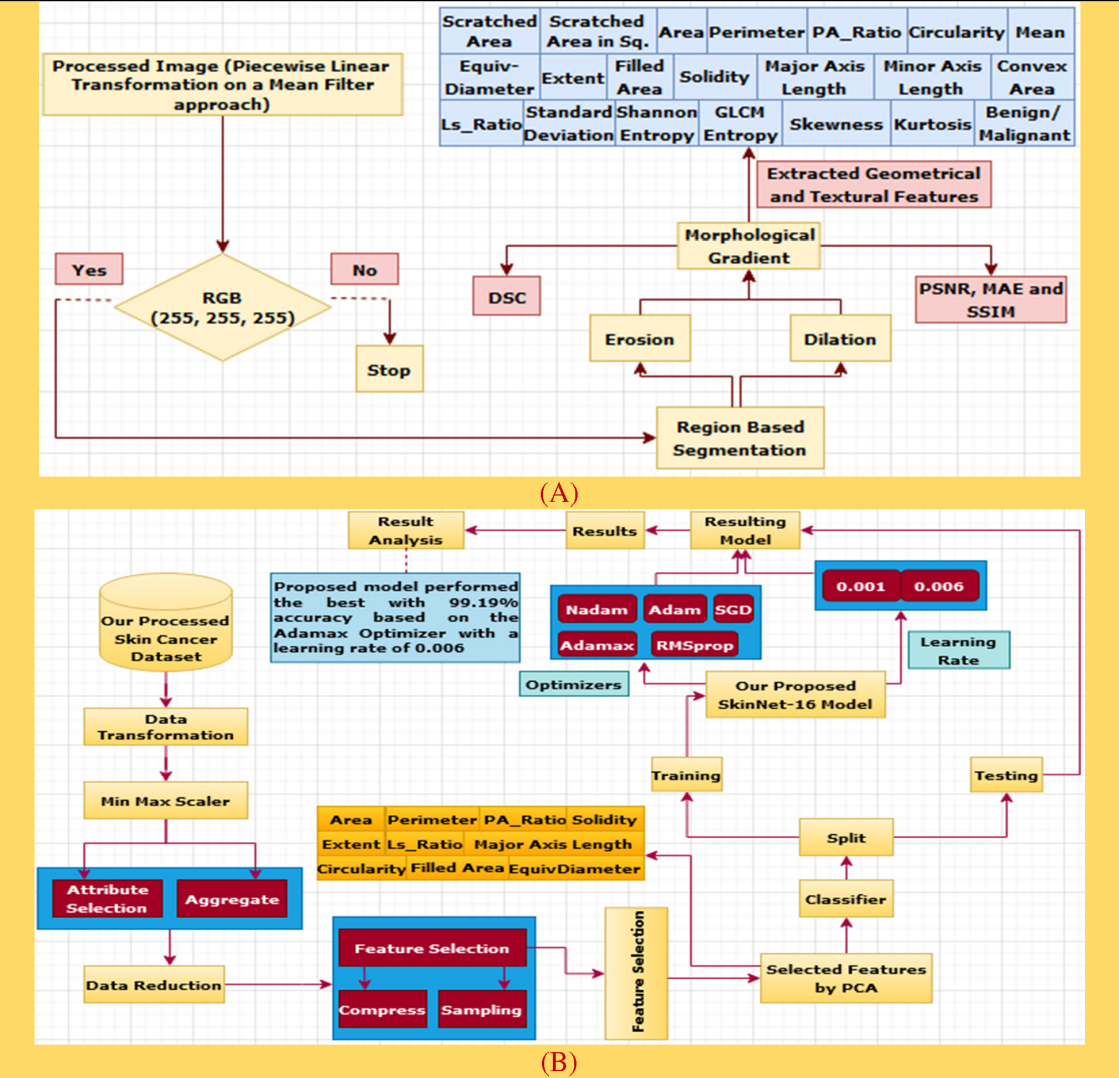
**(A)** The process of data construction. **(B)** A performance analysis diagram based on the extracted features of PCA.

Here, before starting the feature extraction process, we selected piecewise linear transformation (PLT) with a mean filter as an initial image. First of all, we converted it into 255, 255, and 255 values and then did region-based segmentation (RBS) that is selected for classifying the pixel values of different objects based on the threshold value (57) on this converted image. Secondly, erosion (E) and dilation (D) (58) are done based on the RBS image. Both erosion and dilation are two fundamental morphological operations; one deals with removing pixels on object boundaries and the other adding pixels to the boundaries. Thirdly, we generated our desired morphological gradient image (59) that is equal to the difference between this dilation and erosion of an image and to ensure the quality of our working image; we then measure PSNR, MAE, and SSIM values with DSC scores. Furthermore, we are able to extract 20 input features based on both geometrical and textural analyses and a benign or malignant output feature. The overall process is depicted in **Figure 6A**.

#### 4.2.1 Region-based segmentation

Region-based features are extracted and used to define different “classes” (60). Most often, region-based segmentation is texture-based. Textures are considered to be instantiations of underlying stochastic processes and analyzed under the assumptions of stationarity and ergodicity hold. This segmentation relies on similarity and homogeneity of the regions (57).

#### 4.2.2 Erosion

Erosion is a fundamental morphological transformation (58) of image processing. It removes pixels on object boundaries, depending on the size and shape of the structuring element used to process the image. The erosion of a binary image f by a structuring element s (denoted $\Lambda -\frac{1}{2}$ produces a new binary image g = fs with ones in all locations (x, y) of a structuring element’s origin at which that structuring element s fits the input image f, i.e., g (x, y) = 1 is s fits f and 0 otherwise, repeating for all pixel coordinates (x, y). The result is depicted in **Figure 5C**.

#### 4.2.3 Dilation

Dilation, another fundamental morphological operation (58), adds pixels to the boundaries of objects in an image. The dilation of an image f by a structuring element s (denoted fs) produces a new binary image g = fs with ones in all locations (x, y) of a structuring element’s origin at which that structuring element s hits the input image f, i.e., g (x, y) = 1 if s hits f and 0 otherwise, repeating for all pixel coordinates (x, y). Dilation has the opposite effect to erosion—it adds a layer of pixels to both the inner and outer boundaries of regions. The result of dilation is shown in **Figure 5D**.

#### 4.2.4 Morphological gradient

A morphological gradient, as shown in **Figure 5E**, is the difference in mathematical morphology between the dilation and the erosion of a given image (59) and digital image processing. It is an image where each pixel value indicates the contrast intensity in the close neighborhood of that pixel. These are the operators which increase the variation of pixel intensity in a given neighborhood. Based on the morphological_gradient, we extract different types of geometrical and textural features from skin cancer images to generate a dataset.

The following table is added to show the correctness of our processed images depending on the MSE, PSNR, and SSIM values. According to **Table 3**, it is evident that the overall results we generated are trustworthy.

**Table 3:** Comparison on PSNR, MAE, SSIM, and DSC for five morphological gradient (MG) images.

Images	PSNR (dB)	MAE (dB)	SSIM (dB)	DSC
Image 4	44.58	0.43	0.99	0.95
Image 7	46.95	0.29	0.99	0.95
Image 17	41.65	0.82	0.98	0.95
Image 38	45.01	0.43	0.99	0.95
Image 56	46.58	0.33	0.99	0.95

As seen in **Table 3**, the similar numbers of images that have been evaluated to compute the PSNR, MAE, and SSIM results are taken to calculate the dice coefficient scores. Averaging the above values generated 94%, which recommends that applying the proposed methods on our collected images did not affect the skin lesions.

#### 4.2.5 Feature extraction techniques

##### 4.2.5.1 Constructed dataset description

Good data are essential (52) to get a robust result with deep learning techniques. We choose two publicly available datasets downloaded from Kaggle (26) using the preprocessing methods which have been described in the previous section. In the case of the ISIC archive, all the images are recorded in a.csv file with 20 input features, where 1,800 records were from benign and 1,497 from malignant cases. Similarly, the second dataset used in the study is the HAM10000 dataset which has a total of 1,954 images in each benign and malignant class. Then, all the cases are recorded in a.csv file with 20 input features. The 20 attributes of this dataset are used as diagnosis inputs, whereas the “prediction” attribute is selected as an output.

##### 4.2.5.2 Geometrical feature extraction

Geometric features are the features of objects constructed by a set of geometric elements (61) like lines, points, curves, spheres, or surfaces. These features might be corner features, edge features, blobs, ridges, and salient point’s image texture and so on. These can be detected by feature detection methods.

Based on the relation between skin cancers and the locations and shapes of the lesions, it is believed that geometrical features (62) such as {Scratched Area (SA), Scratched Area in sq. Microns (SAS) (63), Area (A), Perimeter (P), P/A Ratio (PR), Major Axis Length (MaAL), Minor Axis Length (MiAL), LS Ratio (LR), Solidity (S), Circularity (C) (64), Filled Area (FA), Extent (Ex), EquivDiameter (E) and ConvexArea (CA)} are very important features for skin cancer detection (see **Table 4**).

**Table 4:** A detailed explanation of geometrical features.

Attributes	Attribute description	Values	Normalized values
**Scratched area**	Scratches are areas of damaged pixels on the surface of the skin. A scratch is a particular surface damage which does not penetrate the lower tissues.	1,117.44 cm^2^	0.8846 cm^2^
**Scratched area in sq. microns**	A measurement of scratched area equal to one micron length by one micron width.	226.28 cm^2^	0.8846 cm^2^
**Area**	The total of all pixels (p) of the segmented nucleus (n).	1327.57 cm2	1 cm^2^
**Perimeter**	The nuclear envelope length is computed as a polygonal length approximation of the boundary (B).	23.71 cm^2^	0 cm^2^
**PA_ Ratio**	It is measured by the degree to which the perimeter of the boundary (B) is the exposed area ratio of the boundary (B).	0.0179 cm	0 cm
**Solidity**	Also known as convexity. The proportion of the pixels in the convex hull that is also in the object. Computed as Area/ConvexArea.	2.2196 cm	0.4066 cm
**EquivDiameter**	Diameter of a circle with the same area as the region, returned as a scalar. Computed as sqrt (4*Area/pi).	41.1135 cm	0 cm
**ConvexArea**	Number of pixels in “ConvexImage”, returned as a scalar	598.117 cm	0.1851 cm
**Circularity**	A measure of circularity (area-to-perimeter ratio) which excludes local irregularities can be obtained as the ratio of the area of an object to the area of a circle with the same convex perimeter: circularity = 4π*area/(convex perimeter)2	29.6843 cm	1 cm
**Extent**	The ratio of pixels in the region to pixels in the total bounding box, returned as a scalar. Computed as the Area divided by the area of the bounding box.	0.0005 cm	0.6667 cm
**FilledArea**	Number of on pixels in FilledImage, returned as a scalar.	23.0981 cm	0.4537 cm
**Minor axis length**	It is computed as the length of the minor axis of an ellipse having the same second moments as the region.	3.6978 cm	0.2002 cm
**Major axis length**	It is computed as the length of the major axis of an ellipse having the same second moments as the region.	6.3934 cm	0.0684 cm
**LS_ Ratio**	It is computed as the length ratio of the major axis length to the minor axis length of the equivalent ellipse of the lesion.	1.7289 cm	0.2540 cm

###### Normalized value

Image normalization is a process (65) in image processing that changes the range of pixel intensity values. Image normalization ensures optimal across data acquisition methods and texture instance comparisons (66). The values from this process are thus normalized. Below, we explain each geometrical feature (67) with its original and normalized values which were used in the proposed method.

##### 4.2.5.3 Textural feature extraction technique

The texture can be defined as a function of spatial variation (68) of the brightness intensity of the pixels. Texture is the main term used to define objects or concepts of a given image, characterized by the spatial distribution of intensity levels in a neighborhood. As a result, textural features are those used to partition images into regions of interest and to classify those regions (69). They provide information on the spatial arrangement of colors or intensities in that image.

Textural feature extraction (62) is important in cancer detection as a tumor can distort a cancerous lesion. There are many techniques for the extraction of textural features (see **Table 5**), namely, Shannon entropy (70), gray-level co-occurrence matrix (71), entropy (GLCME), mean (72), skewness (S), kurtosis (K), and standard deviation (SD), which are widely used nowadays to detect cancerous skin lesions at an early stage.

**Table 5:** Textural features.

Attributes	Attribute description	Values	Normalized values
**Mean**	Mean value is the sum of pixel values divided by the total number of pixel values.	1.583 cm	0.115566
**Standard deviation**	The standard deviation of gray-scale values, ϵ is the estimate of the mean square deviation of the grey pixel value v(x, y) from its mean volume. It describes dispersion within a local region.	1.213 cm	0.249035 cm
**Shannon entropy**	The Shannon entropy can measure the uncertainty of a random process.	0.0972 cm	0.201357 cm
**GLCM entropy**	A gray-level co-occurrence matrix (GLCM) is a histogram of co-occurring grayscale values at a given offset over an image.	1.1172 cm	0.1649 cm
**Skewness**	Skewness is a measure of symmetry, or more precisely, the lack of symmetry. A distribution, or data set, is symmetric if it looks the same to the left and right of the center point.	0.0613 cm	0.7066 cm
**Kurtosis**	Kurtosis is a measure of whether the data are heavy-tailed or light-tailed relative to a normal distribution.	0.2733 cm	0.3564 cm

### 4.3 Analysis of various classifiers based on the constructed dataset

#### 4.3.1 Overview of the proposed algorithm

First off, the dataset we generated based on the highest PSNR, MAE, and SSIM values is prepared for data transformation. There are several transformation techniques, but we selected Min Max Scaler which is applied to the dataset to keep the data between 0 and 1. Secondly, the PCA algorithm (73), a process of data reduction, is used to reduce unnecessary parameters and improve accuracy. The dataset is then separated into training and testing parts. The training phase for the ISIC and HAM10000 datasets is completed with 80% of the data, while remainders (20%) are then used in the testing phase. Among these training data of the ISIC archive, 1,455 images are randomly chosen from the benign case and 1,183 cases are selected from the malignant class. Similarly, 1,563 images from the HAM10000 dataset are randomly selected in each benign and malignant class. Next, 10% of the training phase data from both datasets is experimented for validation. The DL classifier, SkinNet-16, is applied to check the model performance. The prediction rate of this model has been generated based on five different optimizers along with three different learning rates. The process is depicted in **Figure 6B**.

#### 4.3.2 An overview of data transformation technique

The Min Max Scaler techniques (74) has been deployed on our created dataset (See equation 13).


(13) Min Max Scaler m = (X −Xmin) / (Xmax −Xmin)

where m is the updated value and X the original one. Xmin and Xmax represent the minimum number and maximum number of values.

#### 4.3.3 Data reduction process using a feature engineering algorithm

Feature selection techniques (75, 76) are important for deep learning. This also helps to reduce the execution time. We have applied principal component analysis (PCA) to extract features.

##### 4.3.3.1 Principal component analysis

Principal component analysis is a dimensionality reduction technique (77). It uses an orthogonal transformation to convert a set of related variables into a set of linear uncorrelated variables, where the first principal component has the largest variance. This feature extraction technique generates new features which are linear combination of the initial features. PCA maps each instance of the given dataset present in a d dimensional space to a k dimensional subspace such that k < d. The set of k new dimensions generated is called the principal components (PCs), and each principal component is directed toward maximum variance excluding the variance already accounted for in all its preceding components. Subsequently, the first component covers the maximum variance and each component that follows it covers a lesser value of variance. The principal components can be represented by equation (14).


(14) $${\text{PC}}_{\text{i}}={\text{a}}_{1}\;{\text{X}}_{1}+{\text{a}}_{2}\;{\text{X}}_{2}+\cdots +{\text{a}}_{j}\;{\text{X}}_{j}$$

where PC_i_ — principal component ‘i’; Xj — original feature ‘j’; aj — numerical coefficient for Xj.

μ denotes the mean vector. X has size F × N, where F is the number of dimensions and N is the number of observations. Let us assume that B = 〖XX〗^T and C =〖 X〗^T X. It can be supported that both B and C have the same positive eigenvalues Λ, and assuming that N < F, then the eigenvectors U of Band the eigenvectors V of C are related as U =– $-\frac{1}{2}$ matrix with the eigenvectors as columns, and Λ is a (N-1) × (N−1) diagonal matrix with the eigenvalues. Algorithm 5 for calculating PCA has been added below (78).

**Algorithm 5:** Principal Component Analysis.

**BEGIN** 1. Procedure PCA 2. Compute dot product matrix: ${\text{X}}^{T}\text{X}=\sum_{i=1}^{N}{\left({\text{x}}_{i}-\mu \right)}^{T}\left({\text{x}}_{i}-\mu \right)$ 3. Eigenanalysis: X^*T*^X=V*Λ*VT 4. Compute eigenvectors: $\text{U}=\text{XV}\Lambda^{-\frac{1}{2}}$ 5. Keep specific number of first components: 6. Ud = [u1,…, ud] 7. Compute d features: $Y={\text{U}}_{d}^{T}\text{X}$ **END**

##### 4.3.3.2 Outcomes of the PCA algorithm

**Figure 7:**
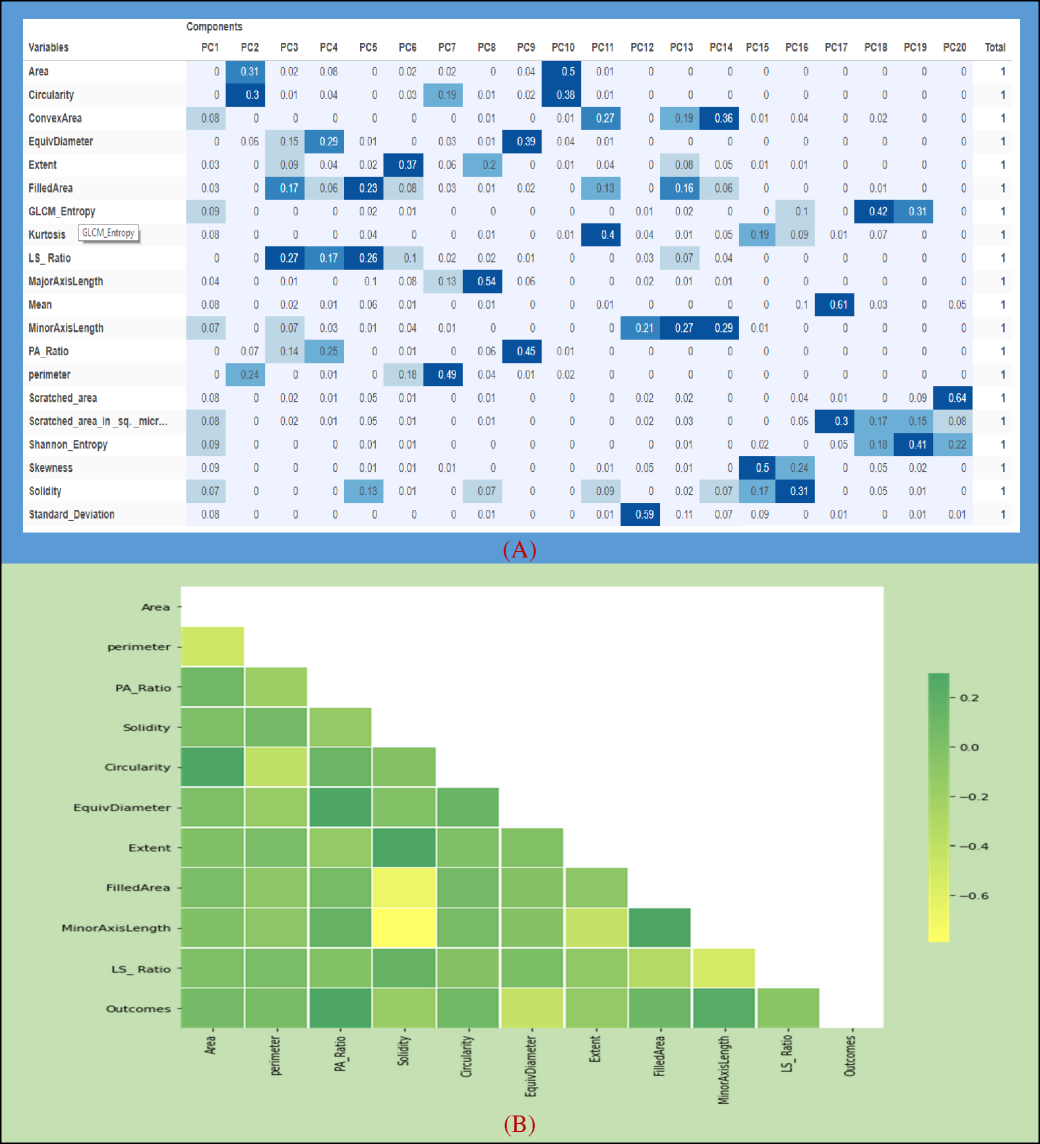
**(A)** Weight components of PCA. **(B)** Histogram plot among the correlated features.

Since the significance from PC1 to PC5 was quite high as compared to other PCA components, we used a summation of the first five components (see **Figure 7A**) based on their total weights and then selected only those features which have a large value for the generated dataset. Later, we selected 10 input features based on this analysis for comparison.

##### 4.3.3.3 Correlation of the selected features

**Figure 7A** indicates the correlation among the evaluated features which have been extracted by the PCA algorithm. The attribute values of the following graph are composed of 10 input features along with an output feature given from 0.2 to -0.6. It can be seen that the correlation between Area and Circularity, EquivDiameter and PA_Ratio, Extent and Solidity, and MinorAxisLength and FilledArea was close to 0.2, whereas a negative correlation (about –0.6) was noticed for FilledArea and MinorAxisLength. (see **Figure 7B**).

#### 4.3.4 Generalized model: no overfitting or underfitting

In case of model overfitting, a model tries to fit the training data entirely and ends up memorizing the data patterns and the noise or random fluctuations (79). These models fail to generalize and perform well in the case of unseen data scenarios, which defeats the model’s purpose. Low bias and high variance are one of the signs of model overfitting.

In our proposed model, the training data samples were adequate and training data were cleaned and made noise-free, which helped the model to generalize the model’s learning. The model was trained with sufficient data for several epochs, and it had low variance.

On the other hand, when the model cannot create a clear mapping between the input and the target variable, underfitting occurs. Under-observing the features leads to a higher error in the training and unseen data samples. It can be detected when the training error is very high, and the model is unable to learn from the training data. High bias and low variance are the most common indicators of underfitting.

In case of our model, there was no chance of uncleaned training data and the dataset was not varied either. It can be stated that no overfitting or underfitting occurred for our proposed model.

#### 4.3.5 Proposed approach for deep learning classification

SkinNet-16 is a model which is based on a convolution neural network (CNN) model. The CNN is one of the most frequently used deep learning classifiers which outperformed its predecessors for the detection of important features (80).

Why CNN has been used: CNNs are the most commonly used deep learning algorithms which outperformed its predecessors for its detection of important features with ease. Its methods have been favorably admitted for numerous imaging classifications (80) for its notable accuracy. In detection of cancerous lesion, we have witnessed this remarkable accuracy while using deep CNN previously. The demonstration of Andre Esteva’s (13), Rehan Ashraf et al.’s (16), Manu Goyal et al.’s (17) studies yielded the performance of CNN as a superior classifier, which made us choose CNN in our approach.

##### 4.3.5.1 SkinNet-16 neural network

**Figure 8:**
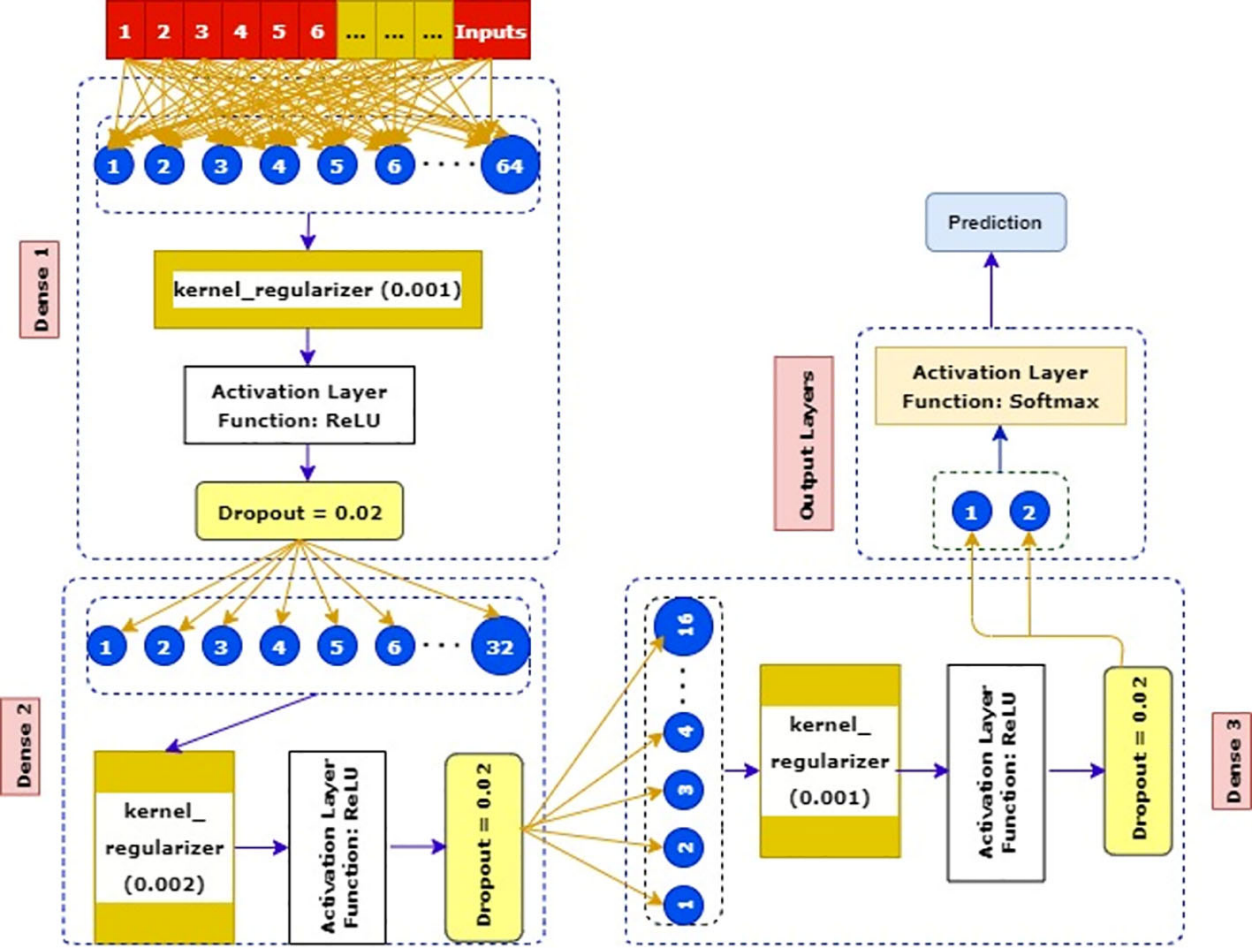
A robust architecture of our pruposed SkinNet - 16 neural network.

SkinNet-16 is a type of convolutional neural network (CNN) and a special case of ANN model (8). The majority of the deep learning algorithms have many layers of artificial neurons to improve accuracy. However, such complex processing requires a larger memory and processing footprint from the hardware. Moreover, the use of optimizers (81) helped us to optimize the learning rate to reduce the losses. For example, Adam computes adaptive learning rates for each parameter which makes it the fastest algorithm to converge to minima, Nesterov-accelerated adaptive moment estimation (Nadam) (81) usually outperforms Adam although it depends on the model, the stochastic gradient descent (SGD) (76) algorithm derivative is computed taking one point at a time requiring way less memory, and Adamax (82) is known for its robustness to gradient update noise and having better numerical stability. We utilized these optimizers and customized this deep learning classifier to get a better result (see **Figure 8**).

We used the training data as inputs, and then we created our first dense layer where 64 neurons were added. After that, a kernel_regularizer which took 0.001 was added as an attribute in this dense layer. Later, one of the most activation layer functions such as ReLU was added. In the second phase of the dense layer, only 32 neurons were taken and connected with 0.002 kernel_regularizer.

Subsequently, similar activation layers and dropout rates of the first dense layer were added here. In the third phase, a kernel regularizer which was 0.001 was connected to a previous dense layer’s dropout rates. Then, the ReLU activation layer and minimal dropout rates (0.002) were added in this layer. In the final phase, the Softmax activation layer along with two output layers benign and malignant was used to get a more robust result.

## 5 Results and discussion

### 5.1 Performance evaluation of different deep learning optimizers

Deep learning is considered to be the most promising field (83) in the detection of skin lesions. It is clearly proved from the past literature described briefly above.

#### 5.1.1 Comparison among all deep learning optimizers based on their testing accuracies for both classes of the ISIC dataset

**Table 6** and **Table 7** describe the results of various optimizers based on the testing accuracy (T_Acc), learning rate (LR), compilation time (CT), validation loss (V_Loss), and validation accuracy (V_Acc). Adamax, Adam, and RMSprop perform very well with an accuracy of around 99% compared to other introduced optimizers in benign class. Besides, Adamax has the lowest 0.001 learning rate and 6.50 validation loss; however, it took a little bit more time (0.0068) compared to the SGD and Nadam optimizers. The validation loss (10.69%) of RMSprop is comparatively higher than the Adam and Nadam optimizers while they have a similar validation accuracy of approximately 98.79%, and in Nadam, the validation loss is the highest among all the optimizers at 12.31%. In terms of malignant class, Adamax only achieves an accuracy of 99% although the validation loss is similar to the benign class.

**Table 6:** A detailed comparison of testing accuracies among all the optimizers with different learning rates, comparison of the optimizers, and optimistic outcomes of various optimizers on the ISIC benign dataset.

Optimizer	LR	CT	V_Loss	V_Acc	T_Acc	SEN	SPE	FPR	FNR	FDR	MSE	RMSE	LL
RMSprop	0.001	0.0126	8.00%	98.79%	99%	98%	98%	2%	2%	2%	1.21%	10.99%	41.78%
	0.006	0.0071	10.69%	98.39%	98%	97%	96%	4%	3%	2%	1.61%	12.70%	55.71%
Adam	0.001	0.0072	9.11%	98.39%	98%	97%	96%	4%	3%	2%	1.61%	12.70%	55.70
	0.006	0.0085	10.22%	98.79%	99%	98%	98%	2%	2%	2%	1.21%	10.99%	41.78%
SGD	0.001	0.0085	6.90%	98.39%	98%	97%	96%	4%	3%	2%	1.61	12.70%	55.70
	0.006	0.0067	6.82%	97.98%	97%	96%	96%	4%	4%	3%	2.01%	14.19%	69.63%
Adamax	0.001	0.0079	7.39%	98.79%	99%	98%	98%	2%	2%	2%	1.21%	10.99%	41.78%
	0.006	0.0068	6.50%	99.19%	99%	99%	99%	1%	1%	1%	0.81%	8.98%	27.85%
Nadam	0.001	0.0075	12.31%	97.98%	97%	96%	96%	4%	4%	3%	2.01%	14.19%	69.63%
	0.006	0.0066	9.79%	98.79%	99%	98%	98%	2%	2%	2%	1.21%	10.99%	41.78%

**Table 7:** A detailed comparison of testing accuracies among all the optimizers with different learning rates, comparison of the optimizers and optimistic outcomes of various optimizers on the ISIC malignant dataset .

Optimizer	LR	CT	V_Loss	V_Acc	T_Acc	SEN	SPE	FPR	FNR	FDR	MSE	RMSE	LL
RMSprop	0.001	0.0126	8.00%	98.79%	97%	96%	96%	4%	4%	3%	1.21%	10.99%	41.78%
	0.006	0.0071	10.69%	98.39%	97%	95%	94%	6%	5%	5%	1.61%	12.70%	55.71%
Adam	0.001	0.0072	9.11%	98.39%	97%	95%	94%	6%	5%	5%	1.61%	12.70%	55.70%
	0.006	0.0085	10.22%	98.79%	97%	96%	96%	4%	4%	3%	1.20%	10.99%	41.78%
SGD	0.001	0.0085	6.90%	98.39%	97%	95%	94%	6%	5%	5%	1.61%	12.70%	55.70%
	0.006	0.0067	6.82%	97.98%	95%	94%	94%	6%	6%	5%	2.01%	14.19%	69.63%
Adamax	0.001	0.0079	7.39%	98.79%	97%	96%	96%	4%	4%	3%	1.21%	10.99%	41.78%
	0.006	0.0068	6.50%	99.19%	99%	98%	98%	2%	2%	1%	0.81%	8.98%	27.85%
Nadam	0.001	0.0075	12.31%	97.98%	95%	94%	94%	6%	6%	5%	2.07%	14.19%	69.63%
	0.006	0.0066	9.79%	98.79%	97%	96%	96%	4%	4%	3%	1.21%	10.99%	41.78%

#### 5.1.2 Comparison of the deep learning optimizers for both classes of the ISIC dataset

**Table 6** and **Table 7** also provide a comparison of the performance of the five optimizers. Adamax gives the best result among all the optimizers. Regarding Adamax, the sensitivity (SEN) and specificity (SPE) are higher than for all other optimizer values at 99% and 99%, respectively, for the benign class. The false-positive (84) rate (FPR), false-negative rate (FNR), and false discovery rate (FDR) (85) are low compared to the other optimizers with values of 1%, 1%, and 1%, respectively. In terms of malignant class, the SEN and SPE rates are above 94% for other four optimizers (Nadam, Adam, SGD, and RMSprop).

#### 5.1.3 Optimistic justification of our proposed techniques for both classes of the ISIC dataset

**Table 6** and **Table 7** depict two types of errors and Log_Loss (LL), along with the learning rate (LR) for the five optimizers. Adamax provided a good performance. This optimizer produces the lowest error rates among all optimizers. For Adamax, the value of mean squared error (MSE) is 0.8065, root mean squared error (RMSE) is 8.9803, and Log_Loss is 27.8539 for the 0.006 learning rate. The other four optimizers produce a comparatively higher error rate. Among these four, SGD provides more error values where Log_Loss is 69.6346 which is similar to Nadam’s value for the 0.001 learning rate. Furthermore, all these results produced are similar in both classes.

#### 5.1.4 Comparison among all deep learning optimizers based on their testing accuracies from the HAM10000 dataset

**Table 8** presents the results of various optimizers based on the testing accuracy (T_Acc), learning rate (LR), compilation time (CT), validation loss (V_Loss), and validation accuracy (V_Acc). Adamax performs adequately with a testing accuracy of 99.19%. Moreover, the learning rate is 0.006 and validation loss is 29.85%. Similarly, the compilation time taken by the optimizer is 0.0044 which is much lower than other optimizers. The validation loss (54.63%) of SGD is comparatively higher than the optimizers. While the optimizers have the almost similar accuracies among them, SGD has the lowest accuracy of 84.87%.

#### 5.1.5 Comparison of the deep learning optimizers from the HAM10000 dataset

**Table 8** delivers a performance comparison of the five employed optimizers. The optimizer Adamax shows sensitivity (SEN) and specificity (SPE) values at 92.5837 and 97.2375, respectively. Subsequently, the false-positive rate (FPR), false-negative rate (FNR), and false discovery rate (FDR) have values of 2.7624, 7.4162, and 2.5188, respectively, which are comparatively much lower than the rest of the optimizers.

#### 5.1.6 Optimistic justification of our proposed techniques from the HAM10000 dataset

**Table 8** portrays mean squared error (MSE) and root mean squared error (RMSE) as well as Log_Loss (LL). The Adamax optimizer produces the lowest error rates where MSE is 5.2564 and RMSE is 22.9268. The Log_Loss is 25.6270 for the 0.006 learning rate. The highest Log Loss of 65.7466 is obtained from the Nadam optimizer at the 0.001 learning rate.

**Table 8:** A detailed comparison of testing accuracies among all the optimizers with different learning rates, comparison of the optimizers, and optimistic outcomes of various optimizers on the HAM10000 dataset.

Optimizer	LR	CT	V_Loss	V_Acc	T_Acc	SEN	SPE	FPR	FNR	FDR	MSE	RMSE	LL
RMSprop	0.001	0.0072	26.83%	90.71%	92.82%	91.89%	93.77%	6.23%	8.10%	6.20%	7.18%	26.79%	50.91%
	0.006	0.0106	33.36%	88.46%	91.79%	92.15%	91.43%	8.57%	7.85%	8.31%	8.21%	28.64%	64.11%
Adam	0.001	0.0084	31.06%	87.82%	90.26%	87.83%	92.95%	7.05%	12.16%	6.72%	9.74%	31.21%	41.37%
	0.006	0.0093	32.01%	86.22%	90.38%	89.66%	91.18%	8.82%	10.34%	8.31%	9.62%	31.01%	55.48%
SGD	0.001	0.0056	54.63%	90.06%	86.28%	84.70%	88.30%	11.69%	15.29%	9.73%	13.72%	37.03%	52.49%
	0.006	0.0087	47.47%	81.73%	84.87%	87.39%	82.5%	17.44%	12.60%	17.88%	15.13%	38.8%	41.97%
Adamax	0.001	0.0091	21.07%	95.51%	94.10%	91.37%	97.25%	2.75%	8.63%	2.56%	5.89%	24.28%	39.65%
	0.006	0.0044	29.85%	93.59%	94.74%	92.58%	97.24%	2.76%	7.42%	2.52%	5.26%	22.93%	25.63%
Nadam	0.001	0.0095	30.35%	91.03%	90.89%	89.74%	92.05%	7.95%	10.26%	8.14%	9.10%	30.17%	65.75%
	0.006	0.0203	31.78%	87.82%	90.51%	91.94%	89.11%	10.89%	8.05%	10.83%	9.49%	30.80%	51.00%

### 5.2 Justification of our proposed model:

Preprocessing is a crucial step of image processing (29) to obtain accurate outcomes. As all the images processed are in 224 × 224 pixels (RGB format), most deep learning algorithms have many layers of artificial neurons to classify the images correctly. Therefore, such complex processing requires a larger memory and processing time. Due to having such complex architecture, machine learning tends to do overfitting. To have this intuition, we convert our images into numerical instances; therefore, the complexity of our work is also minimal. We then propose our SkinNet-16 with a balanced layer. To compute the performance, we create three dense layers where 64, 32, and 16 neurons are added with kernel regularizers which are 0.001. Subsequently, to maintain a balance between our data, we turn off a few parameters with the help of the dropout library. Both dropout and kernel regularizers are basically used to deal with overfitting issues. In the final phase, we use a sigmoid classifier in our study due to binary classification. Consequently, we get a balanced performance from all types of optimizers, and our model produces the results in a minimal time.

### 5.3 Strengths and limitations

#### 5.3.1 Strengths of the study

The study is performed using two prominent datasets that are available publicly. The proposed deep learning model is implemented on both datasets to evaluate its performance. It is observed from the recorded performance measures that the novel model has consistent and high accuracy when applied on both the datasets. Additionally, this model produces the results shown in a minimum amount of time.

#### 5.3.2 Limitations of the work proposed

Skin lesions can be of various types. In the HAM10000 dataset, there are seven types of skin lesions available. However, in the skin, we performed binary classification to detect only the malignant and benign class. At the same time, it is known that any machine learning algorithm requires a large number of data to train the model better. Conversely, the datasets used in the study have a limited amount of image data to train the proposed model.

## 6 Conclusions

In this paper, a novel approach was proposed for detecting skin cancer. First of all, we obtained our dataset from the Kaggle website. Next, we applied the DHR algorithm to remove hairs from the images and applied the rolling ball method to remove background noise, both of which resulted in significant noise reduction. We deployed five image filters to obtain noise-free, unambiguous images on these 3,297 images for further processing where the mean filter exhibited top values in all criteria. The image enhancement technique of piecewise linear transformation (PLT) yielded the best performance after applying all of the five filters based on assessments of PSNR, MAE, SSIM, and histogram analysis to make sure that the image quality was not compromised. The color coding and ROI-based technique facilitated us to select the relevant and accurate region in the image. The morphological operations—dilation, erosion, and morphological gradient—helped us obtain the desired image outcomes, and we considered both geometrical and textural features consisting of 20 different parameters. We observed that PCA was a successful technique in dimensionality reduction in this case and we used it for feature selection resulting in 10 input features for further analysis. We used the SkinNet-16 model which is a modified version of CNN used for skin lesion projection. This prediction has been generated using five different optimizers along with two different learning rates. This intelligent system achieved an overall accuracy of 99.19% on ISIC dataset, based on the Adamax optimizer with a learning rate of 0.006 and more than 98% sensitivity and specificity, which is substantially higher than related works and an indication that demonstrates the efficacy of the proposed system. Thus, it makes a promising approach to detect skin lesions effectively at an early stage.

## Data availability statement

Publicly available datasets were analyzed in this study. This data can be found here: https://github.com/Asif5566/dataextract/blob/7e20ed82adf0077a7670af3f6726aaef0d0336f6/SkinNet-16.zip.

## Author contributions

Conceptualization: PG and RQ; methodology: PG and SA; software: RQ, FS, and KA; validation: SA and MJ; formal analysis: SA, FS, and PG; data curation: SK and RQ; writing—original draft preparation: RQ, KH, and AK; writing—review and editing: SA, RQ, and AK; visualization: SK; supervision: SA and MJ; project administration: AK. All authors contributed to the article and approved the submitted version.

## Conflict of interest

The authors declare that the research was conducted in the absence of any commercial or financial relationships that could be construed as a potential conflict of interest.

## Publisher’s note

All claims expressed in this article are solely those of the authors and do not necessarily represent those of their affiliated organizations, or those of the publisher, the editors and the reviewers. Any product that may be evaluated in this article, or claim that may be made by its manufacturer, is not guaranteed or endorsed by the publisher.
